# Surgical benefit of mandibular morphometric analysis: A new tool to standardize mandibular reconstruction

**DOI:** 10.1371/journal.pone.0240558

**Published:** 2020-11-06

**Authors:** Alice Prevost, Franck Delanoe, Zoé Cavallier, Samuel Muller, Raphael Lopez, Frédéric Lauwers

**Affiliations:** 1 Plastic and Maxillo-Facial Surgery Department, University Hospital Center of Toulouse, Toulouse, France; 2 Clinique de l’Union, Plastic and Maxillo-Facial Surgery Department, Saint Jean, France; 3 Anatomy Laboratory, Paul Sabatier-Toulouse III University, Toulouse, France; Polytechnic University of Marche, ITALY

## Abstract

**Purpose:**

The gold-standard for reconstruction of large mandibular defects is the use of free flaps of vascularized autologous bone with the fibula as the preferred donor site. The use of "custom cutting guides" for this indication is becoming increasingly prevalent. But cost of the procedure averages around 2,500 dollars per patient excluding treatment and entails selection criteria. We think it is possible to standardize mandibular reconstructions from an anatomical mean. The objective of this study was to perform a mandibular morphometric analysis in order to obtain a set of "mean" measurements, which can be used by all surgeons interested in mandibular reconstruction.

**Methods:**

We performed a morphometric analysis consisting of three-dimensional mandibular reconstructions of 30 men and 30 women. Several reference points were set and defined to evaluate specific lengths and angles of interest. We conducted an intra and inter-sexual descriptive analysis of measurements obtained.

**Results:**

We did not identify any major intra-sexual differences within each group. The gonial angle is more open in women and the measurements characterizing the basilar contour are more prominent in men. We did not identify any differences in alveolar region parameters.

**Conclusion:**

The results of this study constitute a morphological tool for surgeons, from bone graft to free flap. These results also confirm us that the use of «custom cutting guides» for mandibular reconstruction may be excessive. It is pertinent to examine the value of "custom made" mandibular reconstructions since the differences observed are of the order of millimeters.

## Introduction

Mandibular reconstruction remains a major morphological and functional challenge.

The currently accepted gold-standard for reconstruction of large mandibular defects is the use of free flaps of vascularized autologous bone with the fibula as the preferred donor site [1, 2].

The use of custom cutting guides for this indication is becoming increasingly prevalent and is widely accepted to significantly reduce the length of time required for the surgical procedures and appears to improve the accuracy of the reconstruction [3–5]. The cost of the procedure averages around 2,500 dollars per patient excluding treatment and entails selection criteria (more complex cases, fragile patients, patients’ ability to assume the cost, etc…). Having used custom cutting guides since 2008, we have noted a number of elements that have led us towards consideration of a possible alternative: using an *universal* cutting guide for fibula free flap micro-anastomosis based on an anatomical average.

The objective of this study is to perform a mandibular morphometric analysis in order to obtain a set of "mean" measurements, which can be used by all surgeons interested in mandibular reconstruction, from bone graft to free flap.

## Materials and methods

### Ethics statements

In this retrospective study, no change to the current clinical practice or randomization was performed. Due to the retrospective nature of this study, it was granted a written exemption from approval by the ethics committee of the Toulouse University Hospital, according to Articles L. 1121–1 paragraph 1 and R 1121–2, paragraph 1 of the French Public Health Code. The authors' Institutional Review Board (IRB) waived the requirement for informed consent. All data were fully anonymized.

The first stage of our work therefore consisted in obtaining a large enough number of three-dimensional reconstructions of non-pathological mandibles (without fracture or tumor process).

### Measurements included in the database

Three-dimensional reconstructions of mandibles were obtained from CT scans (computed tomography scan, Scanner General Electric Medical System, model Optima CT660, slice thickness of 0,6mm). The CT scans of patients included in the study were performed at the emergency room of the University Hospital Center of Toulouse Purpan, France, between January 1^st^, 2017 and June 1^st^, 2017. These images were taken to investigate suspected facial fractures or to assess facial cellulitis. Thirty men and 30 women were included in the analysis [6, 7].

To be included in the study, patients had to:

- Be of adult age, in order to avoid any confounding mandibular developmental phenomena [8, 9].
- Have undergone a CT scan of the facial bone structure in the presence or absence of contrast medium.

Criteria for exclusion were:

- Presence of a tumor or traumatic lesion
- Edentulism (to exclude any confounding secondary bone atrophy)
- Presence of major dental artifacts impeding the quality of the three-dimensional analysis.
- Dental agenesis or the presence of a supernumerary tooth

Images were subsequently exported as de-identified DICOM files (Digital Imaging and Communications in Medicine).

A three-dimensional reconstruction of the mandible based on a DICOM file series was performed using the "OsiriX MD" software. Mandibular reconstructions were subsequently saved as a stereolithographic format (stl).

### Definition of landmarks

**Fig 1:**
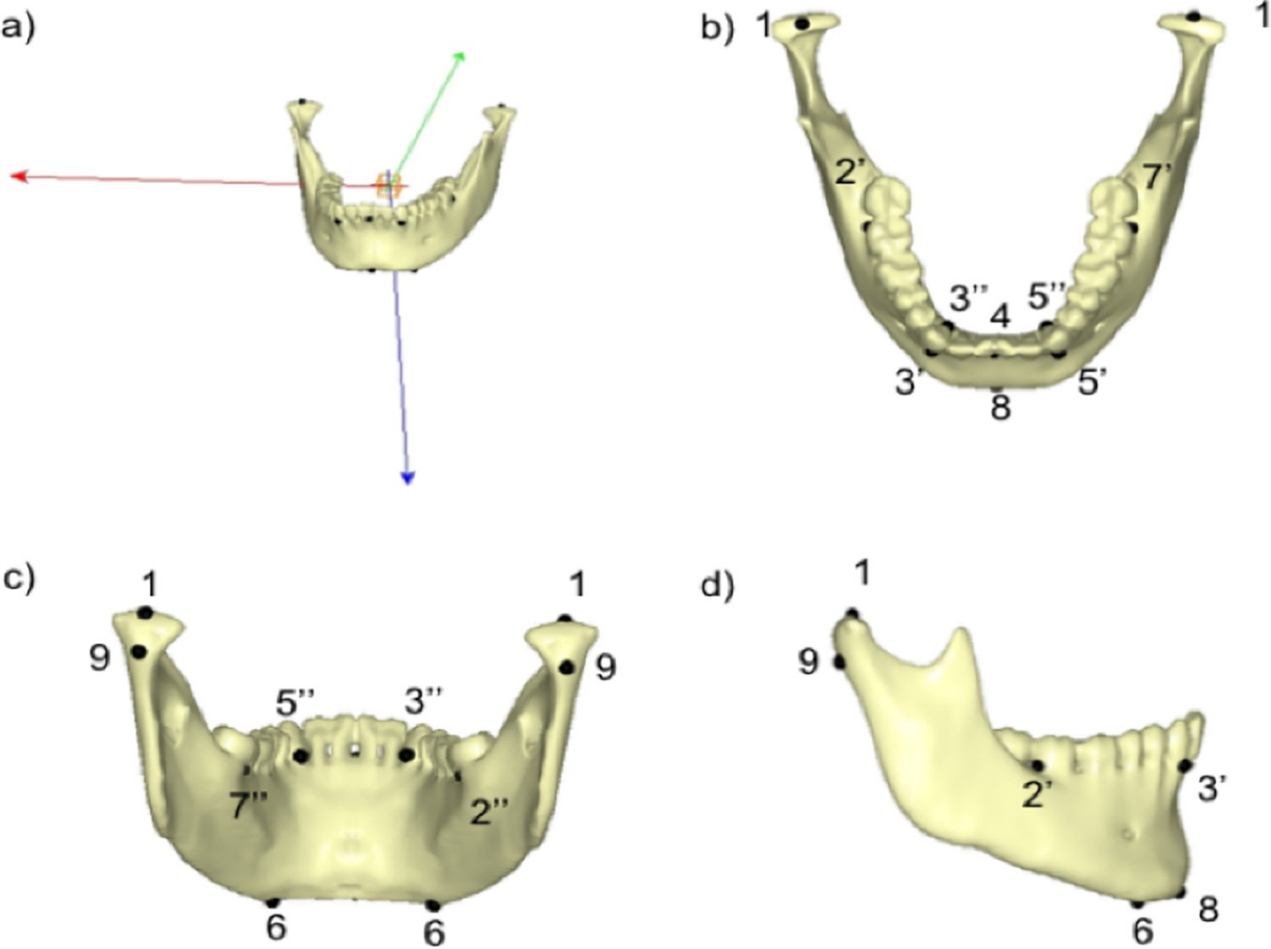
Selected landmarks on the mandible STL file surface. **a:** 3D visualization of an STL file of the mandible in an orthonormal reference frame (Viewbox software). **b-c-d:** LMs are positioned on the surface and are identified by their Cartesian coordinates. 1: Upper condyle. Upper reference point of the condyle.2: Molar 47. Middle of segment 2' (vestibular aspect of the distal surface of 47)-2''(lingual aspect of the distal surface of 47).3: Canine 43. Middle of segment 3' (middle of the vestibular cementoenamel junction (CEJ) of tooth 43)-3''(middle of the lingual CEJ of tooth 43). 4: Inter-incisor reference point. Reference point located in the middle of the CEJ of teeth 31 and 41. 5: Canine 33. Middle of segment 5’ (middle of the vestibular cementoenamel junction (CEJ)of tooth 33) - 5'' (middle of the lingual CEJ of tooth 33) (see below). 6: Basilar projection of the mental foramen. 7: Molar 37. Middle of segment 7’ (vestibular aspect of the distal surface of 37) - 7'' (lingual aspect of the distal surface of 37). 8: Mental protuberance reference point. The lowest reference point and symphysis median. 9: Posterior condyle reference point. Most posterior lying condyle reference point.

**Fig 2:**
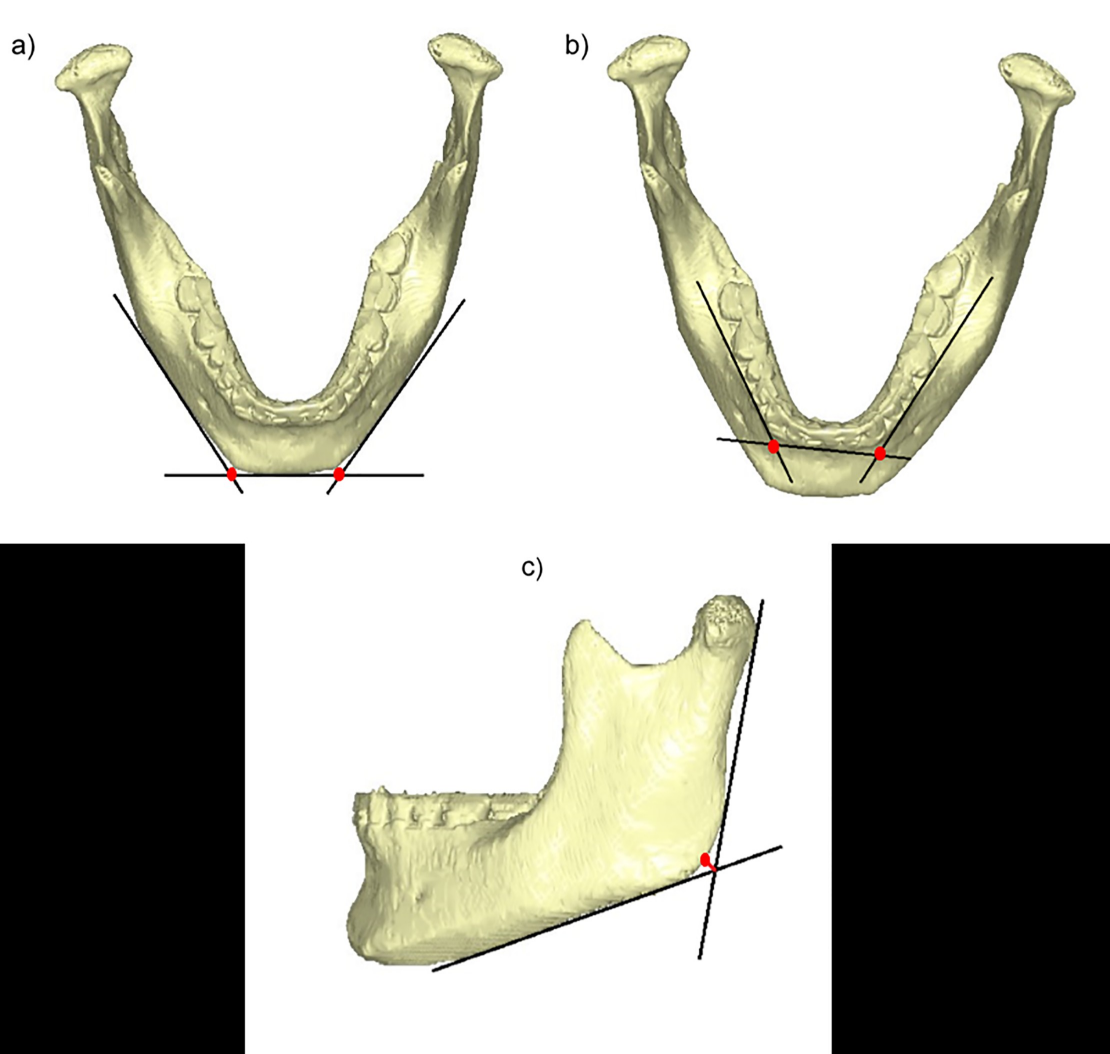
Simplified geometry of the mandible. **a:** the intersection of the tangents to the basilar edge of the symphysis and the basilar edge of the horizontal branch defines the position of the basilar inflexion reference point. **b:** the position of the “alveolar inflexion” reference point is defined by the intersection of the tangents to the alveolar vestibular borders of the incisor/canine and the molar regions. **c:** the gonial angle is defined as the angle between the tangent to the posterior border of the ramus and the tangent to the basilar edge of the mandibular body (horizontal branch). The gonion is the bony projection along the line that bisects the gonial angle.

The geometry of an object can be quantified using a number of different approaches, including contour curves or surfaces [10, 11], but the landmark method was used exclusively for the purposes of the current study. This method relies on the analysis of LM coordinates to capture an object’s geometry. It is essential that these reference points are correctly defined to allow different individual conformations to be compared. The “Viewbox Cephalometric” Software was used to import and process previously obtained STL files. This software allows STL files to be visualized and analyzed within the orthonormal reference frame, by placing landmarks (LM) on the surface of the reconstructed mandibles. Eighteen LMs were placed per patient (Figs 1 and 2).

All measurements were performed by the same observer.

We opted to minimize the number of LMs in order to reduce the complexity of mandibular geometry and focused our analysis on variables relevant to surgical practice.

### Protocol for the analysis of measurements

Previously listed LMs were used to calculate the lengths and angles of interest as defined below (Table 1).

**Table 1:** Metric analysis protocol defining the measurements used for conventional morphometric analysis.

**Bilateral measurements**	
**Gonial angle**	The angle measured between the tangent to the posterior border of the ramus and the tangent to the basilar edge of the mandibular body (horizontal branch).
**Basilar symphysis angle**	The angle between the tangents to the basilar edge of the symphysis and the basilar edge of the horizontal branch
**Canine angle**	The angle between the straight lines of the incisor-canine and canine-molar region
**Vertical posterior dimension**	Distance between the "gonion" and the upper condyle reference point
**Length of dentate region of the horizontal branch**	Alveolar distance between the canine and the second molar
**Basilar length of the horizontal branch**	Basilar distance between the gonion and the “basilar inflection” reference point
**Single measurements**	
**Length of basilar symphysis**	Distance between the two “basilar inflexion” reference points
**Length of the alveolar symphysis**	Distance between the two “alveolar inflexion” reference points
**Symphyseal height**	Distance between the “chin” and “inter-incisor” reference points
**Bi-gonial length**	Distance between the two “gonion” reference points

We have defined a method for calculating these variables with the Viewbox software.

Statistical analyses of the metric variables were performed using version 8.0 of the GraphPad Prism software (GraphPad Software, La Jolla, USA).

The parametric function of our series was tested with a Shapiro-Wilk test. Quantitative data were analyzed using the ANOVA or Friedman test, based on the distribution of variables around the mean.

A p-value less than 5% were considered statistically significant.

To test concordance of the LM reference points, 5 repeat measurements were performed on 10 randomly selected patients. The methodical error was assessed by intraclass correlation coefficient (ICC). Rosner [12] suggested that ICC < 0.4 indicated poor reliability, 0.4 ≤ ICC < 0.75 as fair to good reliability, and ICC ≥ 0.75 as excellent reliability.

## Results

### Population characteristics

The mean age of the female group was 33.66 years (SD: 12.45 years). The mean age of the male group was 31.57 years (SD: 11.14 years). The two groups were comparable, there were no statistically significant differences for this endpoint studied (p = 0.49).

The large standard deviation reflects our intent to include patients of all ages, as long as they fulfilled both the criteria for inclusion and exclusion criteria, since age does not influence the mandibular configuration upon completion of development.

### Intra-sexual variability

ICC ranged from 0.729 to 0.987 for the different measures studied, that indicated an excellent reliability.

### 1. Women

Values obtained for each angle are collated in Table 2.

We observed the following:

- Bilateral measurements are considered symmetrical with a maximal difference of 4,3° tolerated (basilar angle).
- The gonial angle was the most variable inter-individual measurement taken. The standard deviation (and therefore the coefficient of variation) is higher for the measurement of the gonial angle than for any other angle.

**Table 2:** Descriptive statistical analysis of the different angles and length measurements.

	Women		Men	
**Angles** (n = 30)	Mean (degrees) ± SD	Coefficient of variation	Mean (degrees) ± SD	Coefficient of variation
**Right gonial angle**	128,5 ± 8,9	6,9%	125,9 ± 5,7	4,5%
**Left gonial angle**	129,3 ± 8,9	6,9%	125,4 ± 6,1	4,9%
**Right canine angle**	114.6 ± 3.8	3.3%	114.2 ± 4.8	4.2%
**Left canine angle**	114.5 ± 3.6	3.2%	112.9 ± 4.3	3.8%
**Right basilar angle**	122.5 ± 5.1	4.2%	119.2 ± 6.9	5.8%
**Left basilar angle**	118.2 ± 6.5	5.6%	117.1 ± 5.6	4.8%
**Lengths** (n = 30) (centimeters)			(centimeters)	
**Right vertical posterior dimension**	6.0 ± 0.4	6.2%	6.5 ± 0.6	8.7%
**Left vertical posterior dimension**	6.0 ± 0.4	7.0%	6.4 ± 0.5	8.4%
**Length of dentate region of the right horizontal branch**	3.8 ± 0.2	4.2%	4.0 ± 0.3	6.3%
**Length of dentate region of the left horizontal branch**	3.8 ± 0.2	5.1%	4.0 ± 0.3	7.6%
**Basilar length of the right horizontal branch**	7.2 ± 0.6	7.9%	8.0 ± 0.6	7.0%
**Basilar length of the left horizontal branch**	7.5 ± 0.5	6.6%	8.2 ± 0.6	7.2%
**Length of basilar symphysis**	2.5 ± 0.3	12.9%	3.0 ± 0.5	15.6%
**Length of the alveolar symphysis**	2.8 ± 0.2	6.5%	2.9 ± 0.2	7.4%
**Symphyseal height**	2.7 ± 0.3	12.0%	3.1 ± 0.3	8.3%
**Bi-gonial length**	8.8 ± 0.6	6.5%	9.6 ± 0.6	6.6%

All individual length measurements are collated in Table 2.

We observed the following:

- Measurements obtained for respective sides of the same individual are symmetric and display a tighter distribution around the mean than the angle measurements
- Length of the basilar symphysis and symphyseal height are the parameters which varied most (coefficient of variation of 12.9% and 12.0% respectively).

### 2. Men

All angle and length measurements are collated in Table 2:

- For angle, the observed distribution is identical to that determined in the group of women
- As was observed in the female group, the length of the basilar symphysis in the group of men is also the variable with the highest coefficient of variation.

Women-men variables were compared with the sexual dimorphism study.

### Sexual dimorphism of the mandible

As the asymmetry between the two "hemi-mandibles" was expected to interfere with the sexual dimorphism analysis, all subsequent analyses were performed on the mean of each individual’s bilateral variables.

**Fig 3:**
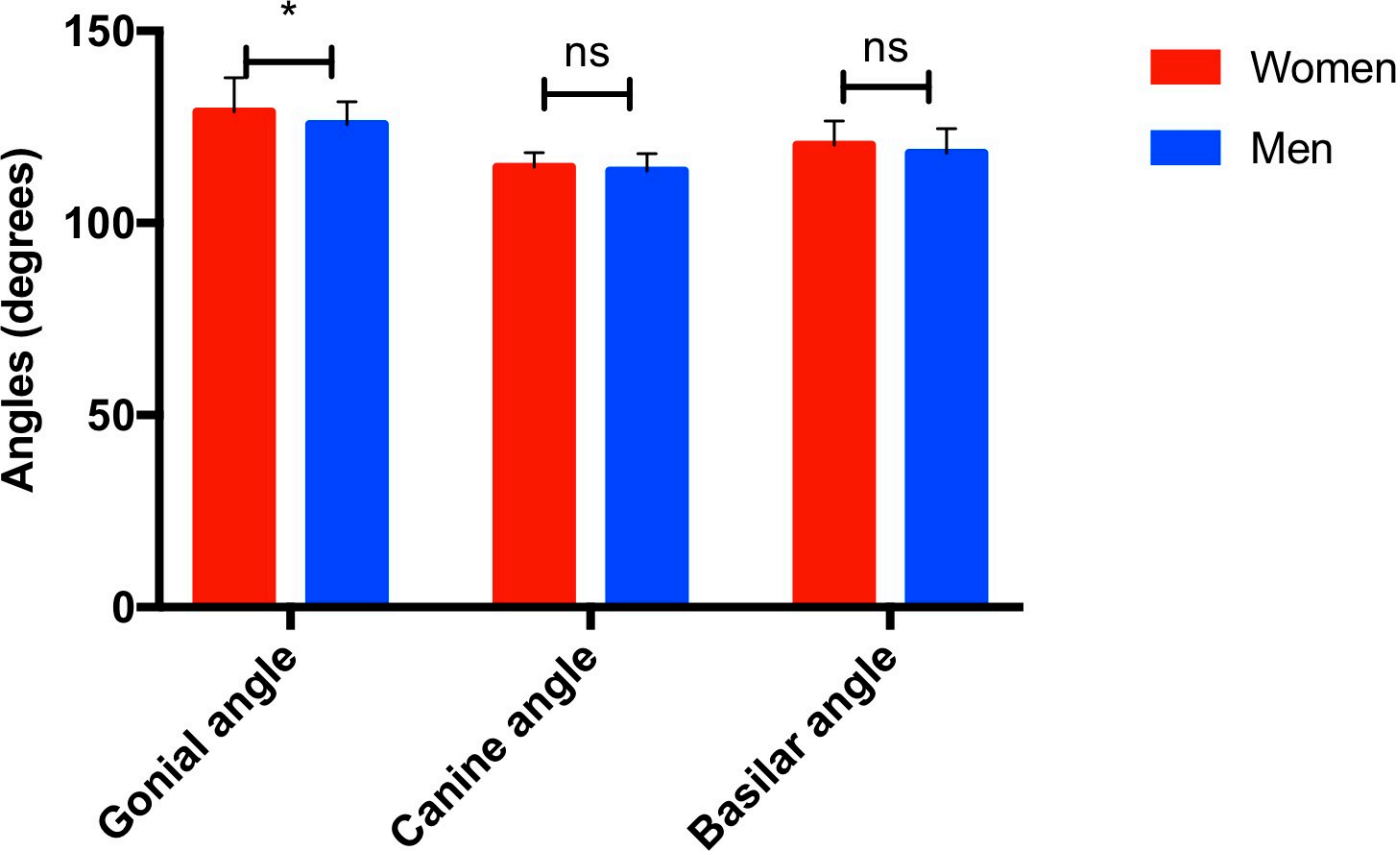
Histogram of the different angle measurements obtained illustrating sexual dimorphism. Gonial angle: women: 128,9° ±8,9°, men: 125,7° ± 5,9°, p = 0,011. Canine angle: women: 114,6° ± 3,7°, men: 113,6° ± 4,6°, p = 0,74. Basilar angle: women: 120,3° ± 6,2°, men: 118,2° ± 6,3°, p = 0,16, n = 30 in each of the groups studied, ns: not statistically significant, *: p <0,05.

With respect to the angles studied, a statistically significant difference between the two groups examined was only observed for the gonial angle: with the angle found to be more obtuse in women (128,9° vs 125,7°, p = 0,011) (**Fig 3**).

Canine and basilar angles were also found to be more open in the female group, although this difference did not reach statistical significance.

**Fig 4:**
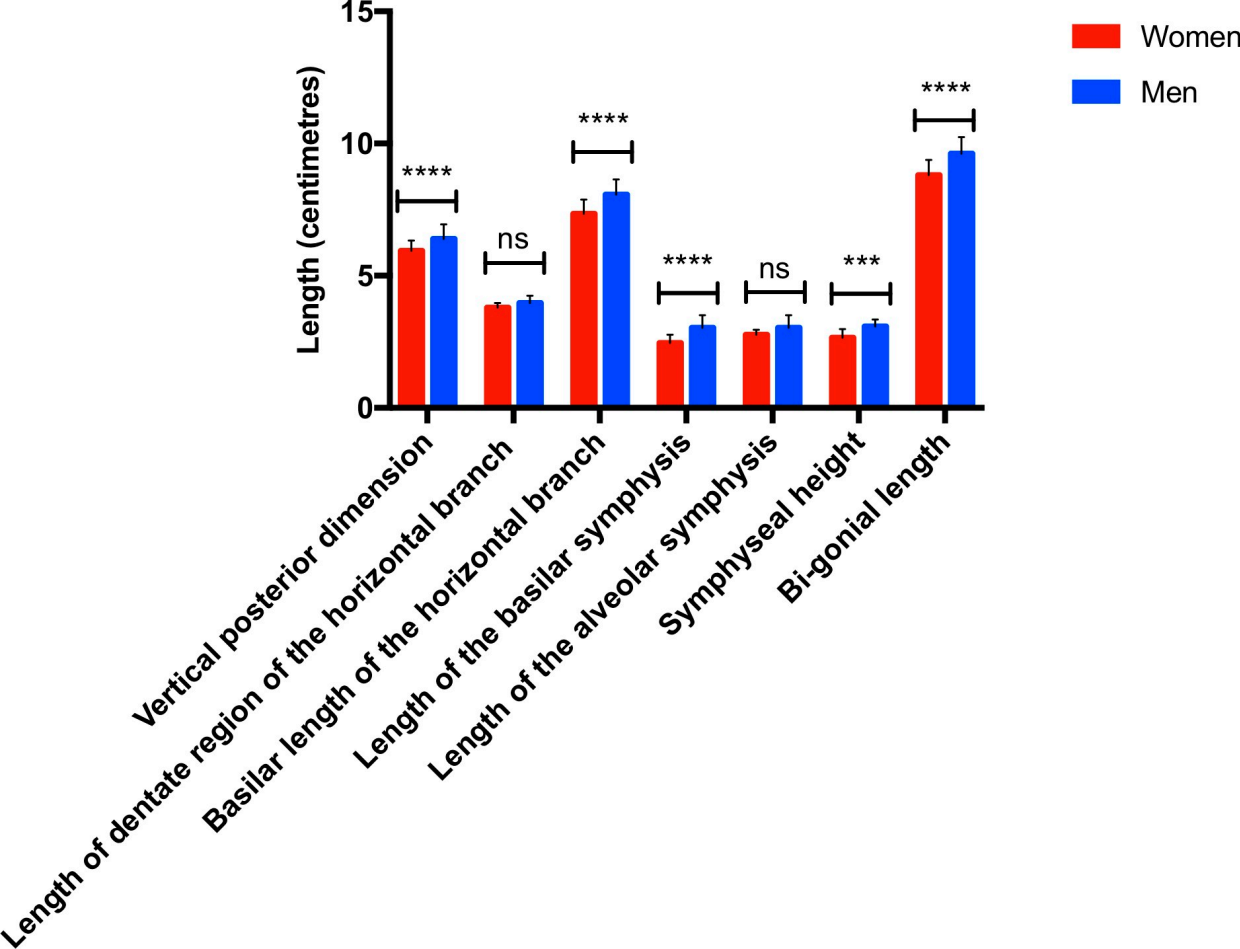
Histogram of the different length measurements obtained illustrating sexual dimorphism. Vertical posterior dimension: women: 5,9cm ± 0,4cm, men: 6,4cm ± 0,5cm, p<0,0001. Length of dentate region of the horizontal branch: women: 3,8cm ± 0,2cm, men: 4,0cm ± 0,3cm, p = 0,21. Basilar length of the horizontal branch: women: 7,3cm ± 0,5cm, men: 8,0cm ± 0,6cm, p<0,0001. Length of the basilar symphysis: women: 2,5cm ± 0,3cm, men: 3,0cm ± 0,5cm, p<0,0001. Length of the alveolar symphysis: women: 2,8cm ± 0,2cm, men: 2,9cm ± 0,2cm, p = 0,90. Symphyseal height: women: 2,7cm ± 0,3cm, men: 3,1cm ± 0,3cm, p = 0,001. Bi-gonial length: women: 8,8cm ± 0,6cm, men: 9,6cm ± 0,6cm, p<0,0001. n = 30 in each of the groups studied. ns: not statistically significant. ***: p <0,001. ****: p <0,0001.

With respect to the different length measurements **(Fig 4),** we observed the following:

- The vertical posterior dimension is statistically significantly longer in the men compared to the women (5,9cm vs 6,4cm, p<0,0001)
- There were no statistically significant differences between the length of dentate region of the horizontal branch (from the canine to the distal surface of the second molar) and between the length of the alveolar symphysis of the women and the men.
- The basilar length of the horizontal branch is statistically longer in the group of men compared to the group of women (8,0cm vs 7,3cm, p<0,0001)
- The basilar symphysis length is statistically longer in the group of men compared to the group of women (3,0cm vs 2,5cm, p<0,0001)
- The symphyseal height length is statistically longer in the group of men compared to the group of women (3,1cm vs 2,7cm, p = 0,001)
- The bi-gonial length is statistically longer in the group of men compared to the group of women (9,6cm vs 8,8cm, p<0,0001)
- There was no statistically significant difference between groups with respect to the length of the alveolar symphysis.

There appears to be a more pronounced sexual dimorphism affecting length compared to angle measurements.

Angles obtained in women also appear to be more open than in men, but the distances measured are larger in men than in women.

## Discussion

Mandibular anatomical studies appear to be of interest to many surgeons in order to facilitate mandibular reconstructions. Anatomical studies already performed, differ from our morphometric study by the methodology used [13].

Some authors have studied anatomical criteria on panoramic radiographs [14], or using others landmarks [15].

Our results are comparable to those in the literature. Nobis and al [15] describe a length "canine-canine" of 26.21mm. Symphysial angle was measured to be 120.39° ± 1.68° for the left side and 120.68° ± 1.44°for the right side.

Our study is the only one to differentiate basal and alveolar parameters.

Concerning our mandible sexual dimorphism analysis, our results are consistent with those reported in the literature [16, 17]. The gonial angle is more open in women and the measurements characterizing basilar contour are more prominent in men. We did not identify any differences in alveolar region parameters.

The overall shape of a man's face can be described as somewhat rectangular, while the lines appear more curved in women [18]. This is a multifactorial observation which is not solely reliant on bone relief. Indeed, masseter muscles are more developed in men [19], while subcutaneous adipose tissue is more prominent in women [20]. These soft tissue factors are very important in mandibular reconstructions, but they are never taken into consideration because they are difficult to evaluate from preoperative imaging data and difficult to control during surgery.

It is pertinent to examine the importance placed on the differentiation between the mandibular reconstruction of men and women since the differences observed are of the order of millimeters. A clear distinction needs to be established between "computer" and "surgical" accuracy by investigating the clinical relevance of identifying a difference in the millimeter range.

Regarding intra-sexual variability, we did not identify any major intra-sexual differences within each group. Indeed, the largest coefficients of variation were obtained with the "gonial angle", "basilar symphysis length" and "symphyseal height” parameters, with standard deviations of the order of one millimeter.

We consider the reconstruction of gonial region and ramus deficits to be a separate issue: the significant intra- and inter-sexual variations in the gonial angle measurement as well as its significant occlusal functional impact would appear to require a reconstructive approach adapted to each individual patient, and therefore does not seem accessible to a universal reconstruction.

The standardization of fibular conformation seems to be judicious for anterior mandibular reconstructions, without compromising postoperative morphological results. Basilar reconstitution seems to be preferred at alveolar reconstitution [21, 22]. Number of fibular osteotomies varies according to the authors, but it is accepted that bone perfusion decreases with number of bone segments [23, 24]. Small bone fragments can compromise vascularization. A length of a minimum of 15 mm is ideal[23]. Ours results respect microvascularization imposed criteria.

## Conclusion

Computer-assisted surgery (CAS) for mandibular reconstruction is booming and indications in maxillofacial reconstruction are more and more numerous.

The use of custom cutting guides for this indication significantly reduce the length of time required for the surgical procedures [25], improves dental restoration, postoperative appearance [26] and appears to improve the accuracy of the reconstruction. Quality of mandibular reconstruction is all the more important as we use a fibular cutting guide and positioning guide [27].

But the cost of the procedure averages around 2,500 dollars per patient. Some authors have developed low-cost, self-made CAD/CAM-guiding system for mandibular reconstruction [28, 29]. These procedures make it possible to avoid the high cost of production, but require too much preoperative planning and printing time.

We thus therefor propose to use an "universal" cutting guide for fibular osteotomies to obtained a symphysis angle of 120° and symphysis length of 25mm. We think it is useless to distinguish men and women’s mandibular reconstruction.

The "universal cutting guide", designed from anatomical means, would expand the indications of guided mandibular reconstructions, allowing a larger number of patients to benefit guided reconstructions, without high cost and manufacturing time. The manufacture of this "universal guide" has already begun. We bring to your attention that studied population concerned only dentate patients. Some parameters may be changed in edentulous patients due to alveolar bone resorption.

In conclusion, results of this study constitute a tool for improving mandibular reconstructions, which remain a major morphological and functional challenge, especially for centers that do not have access to "custom cutting guides".

## Supporting information

S1 File (TXT)

S2 File (TXT)

S3 File (DOCX)

S4 File (XLS)
