# Supplementary figures and images for: Surgical benefit of mandibular morphometric analysis: A new tool to standardize mandibular reconstruction
Source: PLoS One. 2020 Nov 6;15(11):e0240558. doi: 10.1371/journal.pone.0240558 (PMC7647103; doi:10.1371/journal.pone.0240558)

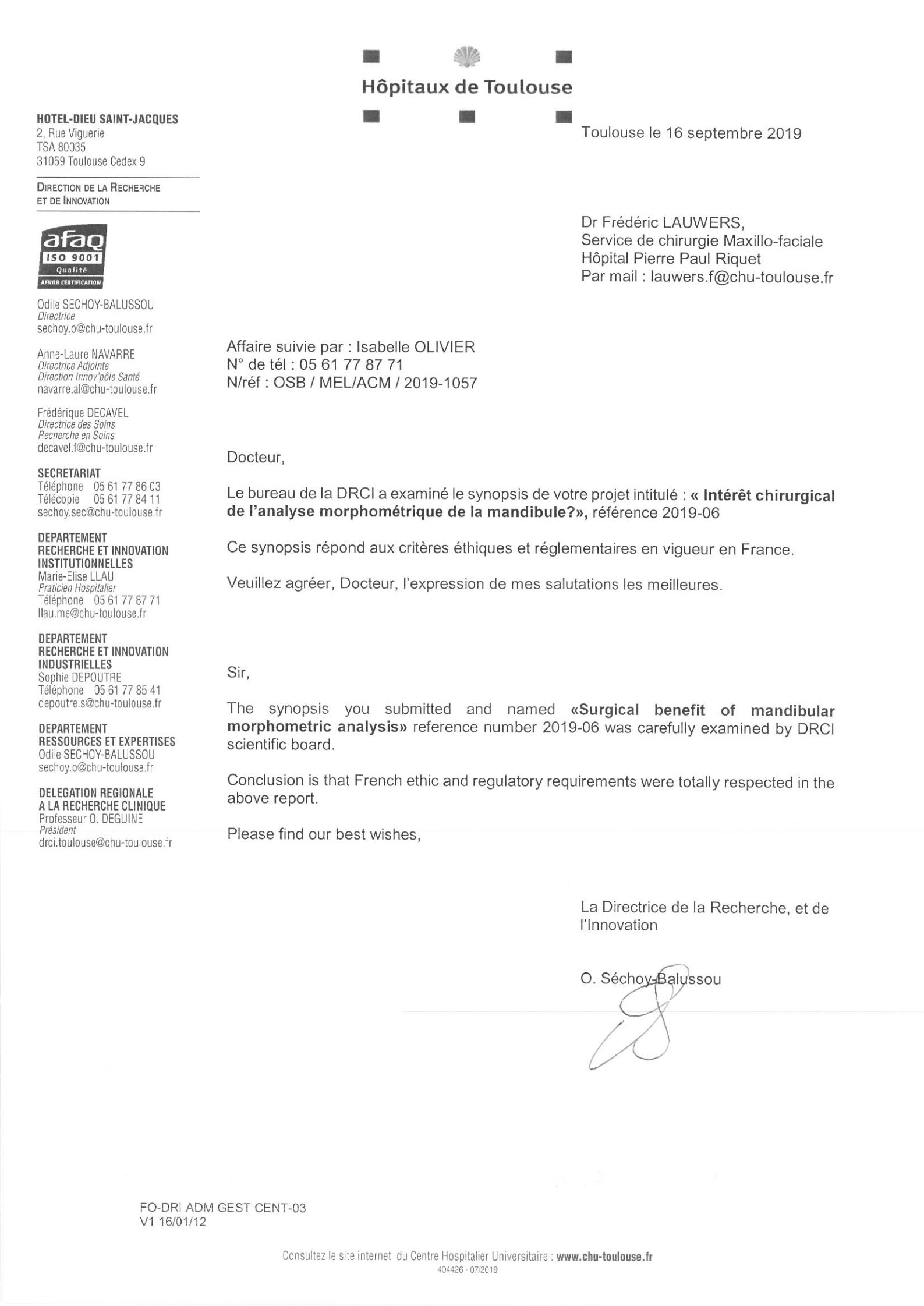

Supplement: S3 File — (DOCX) [file pone.0240558.s003.docx]
